# COVID-19 and HIV: Clinical Outcomes and Inflammatory Markers in a Cohort from a Reference Hospital in Rio de Janeiro, Brazil

**DOI:** 10.3390/v17010091

**Published:** 2025-01-13

**Authors:** Nathalia Beatriz Ramos de Sá, Karine Venegas Macieira, Mariana Rosa Inacio Coelho, Milena Neira Goulart, Marcelo Ribeiro-Alves, Leonardo Azevedo da Silva Rosadas, Kim Mattos Geraldo, Maria Pia Diniz Ribeiro, Sandra Wagner Cardoso, Beatriz Grinsztejn, Valdiléa G. Veloso, Andressa da Silva Cazote, Dalziza Victalina de Almeida, Carmem Beatriz Wagner Giacoia-Gripp, Fernanda Heloise Côrtes, Mariza Gonçalves Morgado

**Affiliations:** 1 Laboratório de AIDS & Imunologia Molecular, Instituto Oswaldo Cruz (IOC), FIOCRUZ, Rio de Janeiro 21040-360, Brazil; karinevenegas94@gmail.com (K.V.M.); marianarosa99b@gmail.com (M.R.I.C.); milenanggoulart@gmail.com (M.N.G.); andressacazote@gmail.com (A.d.S.C.); dalziza@ioc.fiocruz.br (D.V.d.A.); carmembg@ioc.fiocruz.br (C.B.W.G.-G.); fheloise@ioc.fiocruz.br (F.H.C.); 2 Laboratório de Pesquisa Clínica em IST e AIDS, Instituto Nacional de Infectologia Evandro Chagas (INI), FIOCRUZ, Rio de Janeiro 21040-360, Brazil; marcelo.ribeiro@ini.fiocruz.br (M.R.-A.); leonardo.azevedo@ini.fiocruz.br (L.A.d.S.R.); kim.geraldo@ini.fiocruz.br (K.M.G.); mariapia.diniz@ini.fiocruz.br (M.P.D.R.); dra.wagner@gmail.com (S.W.C.); beatriz.grinsztejn@gmail.com (B.G.); valdilea.veloso@gmail.com (V.G.V.)

**Keywords:** COVID-19, HIV-1 infection, cytokines, inflammation

## Abstract

Background: Severe COVID-19 presents a variety of clinical manifestations associated with inflammatory profiles. People living with HIV (PLWH) could face a higher risk of hospitalization and mortality from COVID-19, depending on their immunosuppression levels. This study describes inflammatory markers in COVID-19 clinical outcomes with and without HIV infection. Methods: We analyzed 112 inpatients of the Hospital Center for COVID-19 (INI/FIOCRUZ), including 22 cases of COVID-19 in PLWH (COVID/PLWH group). Plasma samples were tested for a panel of 15 cytokines by Luminex. Sociodemographic, clinical, and laboratory data were collected from patients’ clinical records. Results: COVID-19 individuals were stratified according to the WHO clinical severity profiles at hospitalization. Significant differences in clinical scores, symptoms (coughs), and the occurrence of HIV infection were found among the groups. Clinical blood parameters and plasma cytokines were analyzed among COVID-19 groups with distinct severity profiles. Critical COVID-19 cases showed higher levels of inflammatory markers (Bilirubin, D-dimer, PCR, and urea, as well as IL-8, IL-10, TNF-α, INF-α, IL-1β, IL-17A, IL-23, IL-6) than moderate and severe groups. The COVID/PLWH group had lower CD4 counts (64 cells/mm^3^) and cytokine levels than other COVID-19 patients. Conclusions: Overall, critically ill COVID-19 patients exhibited heightened inflammatory responses, while COVID/PLWH demonstrated unique immunological characteristics without increased mortality risk.

## 1. Introduction

In recent years, new coronaviruses have periodically emerged in different geographical regions, posing a threat to global public health [1]. SARS-CoV-2 emerged at the end of 2019 in Wuhan, China, and spread worldwide, being responsible for a pandemic that affected around 776.5 million individuals globally, with more than 7 million deaths by October 2024. From 2021, with the implementation of an extensive vaccine program worldwide, a significant reduction in deaths due to COVID-19 has been observed [2].

SARS-CoV-2 infection can generate a range of clinical presentations, from asymptomatic, mild, and moderate to more severe forms requiring hospitalization, respiratory failure, and multi-organ involvement, culminating in cure or death. Severe disease is characterized by an unregulated release of cytokines, also called cytokine storm, lymphopenia, deregulation of lymphocyte subsets, and acute lung injury, which can rapidly progress to severe acute respiratory syndrome, disseminated intravascular coagulation, multiple organ failure, and death. Several factors, such as age, comorbidities, and an extensive inflammatory response, may influence the progression of the disease; COVID-19’s progression is still very unpredictable, requiring an urgent need to identify biomarkers to guide the clinical management of COVID-19 [3]. Previous studies have already shown that individuals with COVID-19 had high levels of some inflammatory mediators in more severe cases of the disease, such as C-reactive protein, D-dimer, bilirubin, urea, and IL-1β, IL-6, IL-8, IL-10, IL-18, IFN, TNF-α cytokines [4,5,6,7].

In a prospective study carried out by our group, Perazzo et al. (2022) reported a high hospital mortality rate (27%) in patients hospitalized from June 2020 to March 2021, before the introduction of COVID-19 vaccination in Brazil. These deaths were associated with advanced age, the need for significant ventilatory support, and high severity scores on hospital admission [8]. Several comorbidities, including obesity, diabetes, hypertension, cardiovascular diseases, and chronic kidney disease, have been identified as risk factors associated with severe COVID-19 outcomes [8,9].

Although COVID-19 vaccines have effectively reduced morbidity, hospitalization, and mortality, deaths due to severe manifestations of SARS-CoV-2 infection remain. Depending on the immunosuppression levels, a higher risk of hospitalization and mortality have been described for COVID-19 in people living with HIV (COVID/PLWH), mainly in those without antiretroviral therapy (ART) and with low CD4^+^ T cell count [10,11]. The reasons for the worse prognosis in PLWH are still uncertain. However, some studies showed that chronic immune activation and persistent inflammation are among them [12,13,14,15].

The aim of this study was to describe the clinical outcomes and inflammatory profile in SARS-CoV-2 infected individuals with distinct WHO clinical profiles at hospital admission and PLWH with COVID admitted before COVID-19 vaccination in Brazil. By identifying potential biomarkers, it seeks to deepen our understanding of mechanisms driving COVID-19 severity.

## 2. Materials and Methods

### 2.1. Patient Enrollment and Study Design

This study is nested in the RECOVER-SUS study [NCT04807699], which is a prospective multicenter cohort study that includes participants with SARS-CoV-2 infection who were hospitalized due to COVID-19 at the “Instituto Nacional de Infectologia Evandro Chagas” of the “Fundação Oswaldo Cruz” (INI/FIOCRUZ). Patients were 18 years or older with confirmed SARS-CoV-2 infection, with clinical presentation defined according to the WHO COVID-19 severity classification as moderate, severe, and critical COVID-19 within the first 24 h of hospitalization, detailed elsewhere [16,17,18], who were enrolled in the RECOVER-SUS cohort from June 2020 to March 2021. During this period, 451 were included in the cohort and accepted to participate in the laboratory studies, and among them, 22 were people living with HIV (PLWH). Details regarding patient eligibility, enrollment, inclusion/exclusion criteria, and the study design of the RECOVER-SUS clinical cohort study have been previously described [16,17,18]. For the present study, we analyzed a subgroup of plasma samples from 134 hospitalized individuals of this cohort, including 22 PLWH.

This study was approved by the Ethics Committee of the Instituto Nacional de Infectologia Evandro Chagas (INI)/Fundação Oswaldo Cruz (FIOCRUZ), Rio de Janeiro, Brazil, under CAAE 32449420.4.1001.5262. All participants or their legal representatives signed an informed consent form before enrollment in the study. All methods were performed according to the guidelines and regulations.

Demographic and clinical data and plasma EDTA samples were collected at the study entry visit (baseline). Skin color was self-declared following the classification system employed by the Brazilian Institute of Geography and Statistics (IBGE) [19].

The following clinical blood parameters were recorded from patients’ records: leukocyte, lymphocyte and platelet counts, creatinine, amylase, lipase, alanine aminotransferase (ALT), aspartate aminotransferase (AST), total bilirubin, D-dimer, C-reactive protein (CRP), erythrocyte sedimentation rate (ESR), pro-calcitonin, ferritin, troponin, hemoglobin A1c (HBA1C), prothrombin time activity (PTA), activated partial thromboplastin time (PTT), and urea. For the COVID/PLWH group, the absolute CD4^+^ and CD8^+^ T cell count, as well as the HIV-1 plasma viral load, were also obtained from the hospital records.

### 2.2. Clinical Profiles at Presentation

The clinical presentation of the individuals included in this study was defined according to the WHO COVID-19 severity classification as moderate, severe, and critical COVID-19 within the first 24 h of hospitalization as detailed elsewhere [17,20].

### 2.3. HIV Diagnosis

To identify/confirm cases of HIV infection, plasma samples from individuals included in the cohort were screened for HIV infection using a rapid test (4th generation RDT Alere^TM^ HIV Combo—Abbott Alere, Waltham, MA, USA).

### 2.4. Plasmatic Inflammatory Markers

Plasmatic levels of IL-1β, IL-8, IL-10, IL12p70, IL-17A, IL-17F, IL17AF, IL-18, IL-23, IL-33, IP10, TNF-α, IFN-α, and IFN-γ were measured at study entry (baseline) using a customized ProcartaPlex multiplex immunoassay (Invitrogen, Waltham, MA, USA) according to the manufacturer’s instructions and a MAGPIX reader (Luminex Corp, Austin, TX, USA). IL-6 plasma levels were assessed using an ELISA kit (Invitrogen, USA).

### 2.5. Statistical Analysis

For descriptive analysis of the individuals classified according to the WHO COVID-19 severity classification and for those classified according to their HIV-1 co-infection profile, the categorical variables were expressed as absolute and relative frequencies, while continuous variables were as medians and interquartile ranges (IQRs). Either Kruskall–Wallis or Mann–Whitney U tests (continuous–numerical), respectively, or chi-square tests (categorical–nominal variables) were used to compare groups. Multiple linear fixed effects models were used for inferential analysis. Confounding variables were considered for data adjustment, such as sex, age, and days since the onset of symptoms. Variables studied were log-transformed when suitable. Mean marginal values were estimated by model parameters, where all but the exposition/group effect in the systematic component of the linear multiple models were maintained at their mean values or equal proportions. Contrasts were constructed from these mean marginal effects. The Tukey Honest Significant Difference (HSD) method corrected *p*-values for the number of comparisons, where applicable. Similarly, correlation analyses were conducted using Pearson’s coefficients (ρ) after adjustments for the confounding variables. Correlations were further categorized as weak (0.3–0.4), moderate (0.4–0.7), and strong (0.7–1); *p*-values < 0.05 were considered statistically significant. R software version 4.1.1, packages ‘lm’ and ‘emmeans’ and their dependencies were utilized.

## 3. Results

### 3.1. Sociodemographic and Clinical Characteristics of the Cohort

The COVID-19 individuals included in this study (n = 134) were stratified according to the WHO clinical severity profiles at hospitalization. Sociodemographic and clinical characteristics at hospital admission are depicted in Table 1.

Data from 134 participants were analyzed, 80 (59.7%) individuals were discharged, and 54 (40.3%) died during the hospital stay. Overall, most of the participants were male 74 (55.2%), with 15 (50%) presenting moderate disease (WHO 4–5 groups), 38 (56.7%) severe disease (WHO 6–8 groups), and 19 (59.4%) critical disease (WHO 9–10 groups). Overall, the mean age was 58 years (IQR = 21.3), with no differences among the three WHO severity groups. Most of these individuals (n = 99; 76.7%) were classified as having WHO severity scores between 6 and 10 (severe/critical). Also, the frequencies of the individuals according to gender, skin color, age, and schooling did not differ among the groups. Several common symptoms were observed in the clinical presentation of COVID-19 individuals (Table 1), but only cough (*p* = 0.025) showed statistically significant differences among the groups.

### 3.2. Sociodemographic and Clinical Characteristics of the COVID/PLWH Group

We further divided our study cohort into two groups according to HIV status: 112 individuals with COVID-19 without HIV (COVID-19 group) and 22 individuals with COVID-19 living with HIV (COVID/PLWH group) to compare their sociodemographic and clinical characteristics, as depicted in Table 2.

Most of the COVID/PLWH individuals were male (59.1%). It is of attention that their median age was much lower than those with only COVID-19 (44 years [IQR = 17.84] vs. 62.61 [IQR = 19.37]; *p* < 0.001). No significant differences in gender, skin color, and schooling were observed between these groups. Overall, 116 individuals (86.6%) required some use of oxygen support at clinical presentations, with significant differences between the COVID-19 and COVID/PLWH groups (91.1% vs. 63.6%; *p* = 0.002). Regarding comorbidities, only systemic arterial hypertension (*p* = 0.012) and active tuberculosis (*p* = 0.001) had differences between the two groups. Among the symptoms, only dyspnea showed a significant difference between the groups (*p* = 0.034), being less frequent in the COVID/PLWH group. Furthermore, no statistically significant differences were found between the COVID and COVID/PLWH groups regarding discharge or death (*p* = 0.862) (Table 2). 

Considering the specific parameters of HIV infection, the state of heavy immunosuppression observed in the randomly identified HIV-positive individuals in our inpatient study group during recruitment is of particular attention. These individuals presented a median CD4^+^ T cell count of 64 cells/mm^3^ (IQR = 239), CD8^+^ T cell count of 514 cells/mm^3^ (IQR = 351), and a viral load of 92,151 copies/mL (IQR = 628,649.75) (Table 2).

### 3.3. Clinical Blood Parameters and Plasma Cytokines Among COVID-19 Individuals with Distinct Severity Profiles

**Figure 1:**
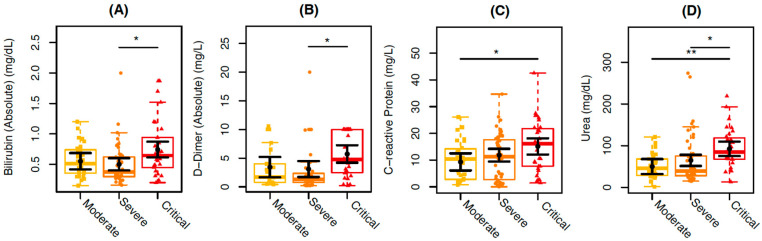
Comparison of clinical blood marker levels among the individuals in the study categorized according to the COVID-19 severity: moderate (in yellow), severe (in orange), and critical (in red). The graphs represent the concentration measures of (**A**) bilirubin, (**B**) D-dimer, (**C**) C-reactive protein, and (**D**) urea. The sampling distributions of the data are represented in the form of a colored box plot. In black, the center circle represents each group’s expected mean marginal effect, estimated from linear fixed effects models including sex, age, and days since the first symptoms as confounding variables. The horizontal bars represent the 95% confidence intervals of the expected mean marginal effects by group. *p*-values were corrected for the number of two-by-two contrast/comparisons by the Tukey Honest Significant Difference (HSD) method. * *p*  <  0.05; ** *p*  <  0.01.

We further analyzed 19 clinical laboratory parameters data of the COVID-19 inpatients included in this study, classified as having moderate, severe, and critical disease independent of the HIV-associated infection. Statistically significant results are shown in Figure 1.

Clinical routine blood markers, such as D-dimer (*p* = 0.0361), bilirubin (*p* = 0.0118), and urea (*p* = 0.0375), show higher mean values of 2.62 mg/L, 0.2 mg/dL, and 27.72 mg/dL, respectively, in individuals with severe COVID-19 compared to those individuals with critical COVID-19. Similarly, C-reactive protein (*p* = 0.0267) and urea (*p* = 0.0027) showed higher mean values of 5.81 mg/L and 42.45 mg/dL, respectively, in individuals with moderate COVID-19 compared to those with critical COVID-19 (Figure 1). No differences among the groups were observed for leukocytes, lymphocytes, platelets, creatinine, amylase, lipase, ALT (alanine aminotransferase), AST (aspartate aminotransferase), ESR (erythrocyte sedimentation rate), procalcitonin, ferritin, troponin, HBA1C (hemoglobin A1c), PTA (prothrombin time activity), and PTT (partial thromboplastin time).

**Figure 2:**
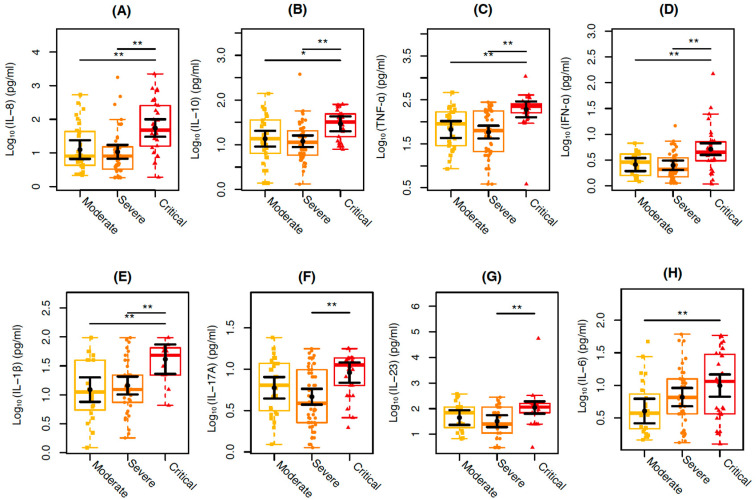
Cytokine plasma levels among the individuals included in the study categorized according to the COVID-19 severity: moderate (in yellow), severe (in orange), and critical (in red). The graphs represent the Log10-transformed concentration measures by multiplex Luminex of (**A**) IL-8, (**B**) IL-10, (**C**) TNF-α, (**D**) IFN-α, (**E**) IL-1β, (**F**) IL-17A, (**G**) IL-23, (**H**) IL-6. The sampling distributions of the data are represented in the form of a colored box plot. In black, the center circle represents each group’s expected mean marginal effect, estimated from linear fixed effects models including sex, age, and days since the first symptoms as confounding variables. The horizontal bars represent the 95% confidence intervals of the expected mean marginal effects by group. *p*-values were corrected for the number of two-by-two contrast/comparisons by the Tukey Honest Significant Difference (HSD) method. * *p*  <  0.05; ** *p*  <  0.01.

Proinflammatory and regulatory cytokine levels were also compared among the moderate, severe, and critical groups independent of the HIV-associated infection. In general, the critical COVID-19 group presented the highest levels for several cytokines, as shown in Figure 2.

The critical COVID-19 group showed, respectively, 338% and 409% higher values of IL-8 (*p* = 0.0030; *p* = 0.0002) in relation to the moderate and severe COVID-19 groups, 115% and 145% of IL-10 (*p* = 0.0191; *p* = 0.0012), 187% and 228% of TNF-α (*p* = 0.0022; *p* < 0.001), 99% and 106% of IFN-α (*p* = 0.0020; *p* = 0.0002), and 236% and 186% of IL-1β (*p* = 0.0060; *p* = 0.0093). IL-17A (*p* = 0.0010) and IL-23 (*p* = 0.0064) showed 96% and 241% higher values, respectively, in individuals with severe COVID-19 compared to those with critical COVID-19, whereas 146% higher values of IL-6 (*p* = 0.0083) was observed in individuals with moderate COVID-19 compared to those with critical COVID-19 (Figure 2). No differences among the WHO clinical groups were observed for IFN-γ, IL-12p70, IL-17A, IL-17F, IL-17AF, IL-18, IL-33, and CXCL10/IP-10 plasma levels analyzed.

### 3.4. Clinical Blood Parameters and Cytokines Markers of COVID-19 and COVID/PLWH Individuals

Regarding laboratory results of all the individuals included in the study, the median (IQR) of leucocytes, lymphocytes, and platelets counts were 10,260 copies/mL (IQR = 7710), 1121 cells/mm^3^ (IQR = 1122), and 258 cells/mm^3^ (IQR = 127.5), respectively. Additionally, median (IQR) levels of D-dimer, C-reactive protein, and ferritin were 1.95 mg/L (IQR = 5.9), 11.05 µg/L (IQR = 13.88), and 525.9 ng/mL (IQR = 743.5), respectively (Supplementary Table S1). In addition, regarding the cytokine results of all the individuals included in the study, the median (IQR) is also shown in Supplementary Table S1.

ALT (*p* = 0.0121) and AST (*p* = 0.0329) showed higher mean values of 35.6 U/L and 20.4 U/L, respectively, in the individuals with COVID-19 group compared with the COVID/PLWH (Figure S1). No differences between these two groups were observed for the COVID-19 major inflammatory markers, such as D-dimer, CRP, and IL-6.

**Figure 3:**
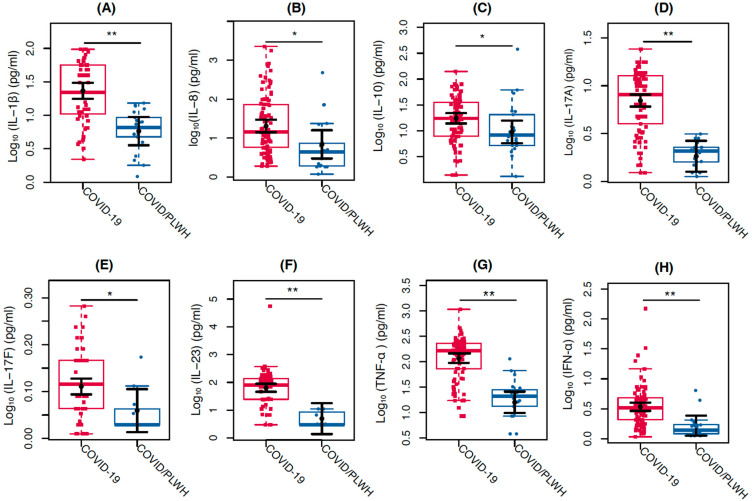
Comparison of cytokine levels between COVID-19 individuals (in red) and COVID/PLWH individuals (in blue). The graphs represent the Log10-transformed concentration measures by multiplex Luminex of (**A**) IL-1β, (**B**) IL-8, (**C**) IL-10, (**D**) IL-17A, (**E**) IL-17F, (**F**) IL-23, (**G**) TNF-α, and (**H**) IFN-α. The sampling distributions of the data are represented in the form of a colored box plot. In black, the center circle represents each group’s expected mean marginal effect, estimated from linear fixed effects models including sex, age, and days since the first symptoms as confounding variables. The horizontal bars represent the 95% confidence intervals of the expected mean marginal effects by group. * *p*  <  0.05; ** *p*  <  0.01.

Regarding cytokines, IL-1β (*p* < 0.0001), IL-8 (*p* = 0.0241), IL-10 (*p* = 0.0373), IL-17A (*p* < 0.0001), IL-17F (*p* = 0.0404), IL-23 (*p* = 0.0003), TNF-α (*p* < 0.0001), and IFN-α (*p* = 0.0010) showed 302%, 196%, 83%, 282%, 13%, 1179%, 637%, and 107% higher values, respectively, in the individuals with COVID-19 group compared with the COVID/PLWH (Figure 3).

### 3.5. Correlations Between the Clinical Laboratory and Cytokines Markers

**Figure 4:**
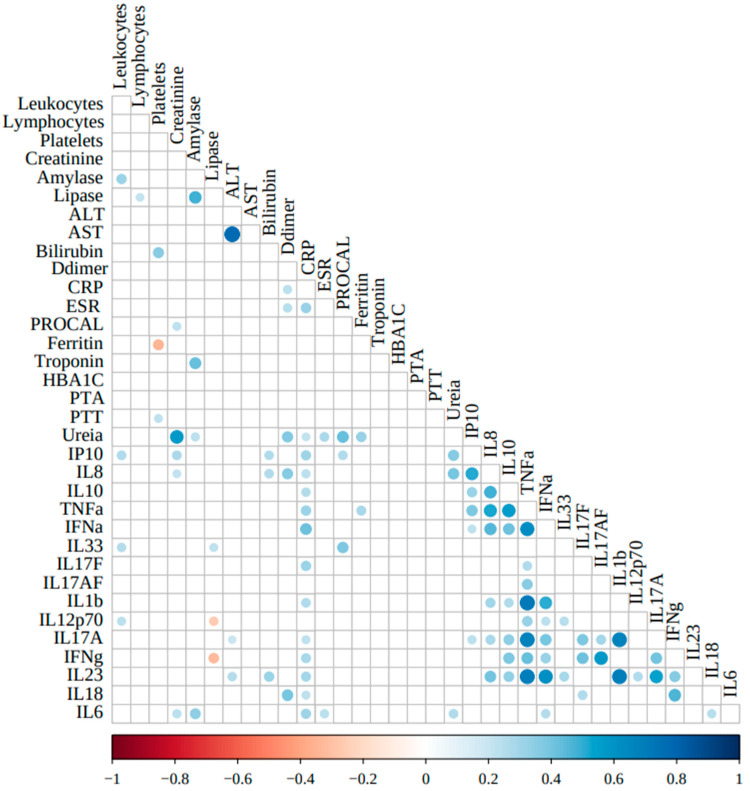
Correlations of current plasma and cells COVID-19 clinical markers and inflammatory and regulatory cytokines evaluated in this study. Inflammatory/regulatory cytokine levels were Log10-transformed. Correlation analyses were conducted using Pearson’s coefficients after adjustments for the confounding variables sex, age, and days since the first symptoms. Abbreviations: ALT: alanine aminotransferase; AST: aspartate aminotransferase; CRP: C-reactive protein; ESR: erythrocyte sedimentation rate; PROCAL: procalcitonin; HBA1C: hemoglobin A1c; PTA: prothrombin time activity; PTT: partial thromboplastin time.

To better understand the role of inflammation markers in COVID-19 inpatient clinical profiles, we correlated the inflammatory/regulatory cytokine levels and the routine clinical laboratory severity-associated markers. Figure 4 depicts the correlations among the parameters analyzed.

Among the cytokines, only IL-1β correlated strongly/positively with TNF-α (ρ = 0.716; *p* < 0.0001). TNF-α also correlated moderately/positively with IL-8 (ρ = 0.529; *p* < 0.0001), IL-10 (ρ = 0.560; *p* < 0.0001), IL-17A (ρ = 0.678; *p* < 0.0001), IL-23 (ρ = 0.698; *p* < 0.0001), IFN-α (ρ = 0.624; *p* < 0.0001), IFN-γ (ρ = 0.422; *p* = 0.003), while IL-1β correlated moderately/positively with IL-17A (ρ = 0.678; *p* < 0.0001), IL-23 (ρ = 0.690; *p* < 0.0001), and IFN-α (ρ = 0.500; *p* < 0.0001).

Among the clinical markers, as expected, the hepatic enzymes ALT and AST correlated strongly/positively with each other (ρ = 0.772; *p* < 0.0001). Also, amylase correlated moderately/positively with lipase (ρ = 0.482; *p* < 0.0001) and troponin (ρ = 0.423; *p* < 0.0001); urea correlated moderately/positively with creatinine (ρ = 0.574; *p* < 0.0001) and procalcitonin (ρ = 0.425; *p* < 0.0001).

No strong correlations were observed among the clinical markers of inflammation, coagulation, glycemic control, hepatic or renal functions, and the selected cytokines analyzed in this study. Only plasma concentrations of C-reactive protein correlated moderately/positively with IFN-α (ρ = 0.413; *p* < 0.0001), while only weakly/positively with CXCL10/IP-10 (ρ = 0.307; *p* = 0.004), TNF-α (ρ = 0.314; *p* = 0.001), IL-17F (ρ = 0.310; *p* = 0.009), and IL-6 (ρ = 0.304; *p* = 0.003).

Regarding correlated moderate/positive among cytokines, we can observe between IL-8 and IP-10 (ρ = 0.512; *p* < 0.0001), IL-10 (ρ = 0.482; *p* < 0.0001), TNF-α (ρ = 0.529; *p* < 0.0001), IFN-α (ρ = 0.454; *p* < 0.0001), and IL-23 (ρ = 0.407; *p* = 0.0004). Also, IL-10 with TNF-α (ρ = 0.560; *p* < 0.0001) and IFN-α (ρ = 0.414; *p* < 0.0001). Also, IFN-α with IL-23 (ρ = 0.617; *p* < 0.0001). Also, IL-17F with IFN-γ (ρ = 0.411; *p* = 0.019); IL-17AF with IFN-γ (ρ = 0.574; *p* = 0.0009); IL-17A with IFN-γ (ρ = 0.403; *p* = 0.003) and IL-23 (ρ = 0.547; *p* < 0.0001); and IFN-γ with IL-18 (ρ = 0.468; *p* = 0.0009).

Weakly/negative correlations were observed only between ferritin and platelets (ρ = -0.349; *p* = 0.0047) and lipase and IFN-γ (ρ = −0.325; *p* = 0.0201).

## 4. Discussion

The main factors that can predict the severity of COVID-19 are not yet fully defined, especially at the laboratory biomarker levels. In addition, immunological and genetic factors that contribute to the “cytokine storm”, one of the major determinants of the severity of COVID-19, have not yet been fully characterized. In this study, we evaluated a panel of cytokines and clinical laboratory markers that might be associated with the severity of COVID-19, as defined according to the WHO clinical classification. During this study, we identified a subset of PLWH within our cohort of hospitalized individuals with COVID-19. This provides a pivotal opportunity to elucidate the modulatory effects of HIV-1 infection on the clinical phenotypes and immunopathological trajectories of COVID-19.

We observed higher levels of bilirubin, D-dimer, C-reactive protein, urea, and plasmatic cytokines, such as IL-1β, IL-6, IL-8, IL-10, IL-17A, IL-23, TNF-α, and IFN-α in individuals with the critical form of acute COVID-19 compared to individuals with the severe and/or moderate WHO clinical forms. In addition, only mild correlations were observed among the plasmatic proinflammatory cytokines and laboratory clinical markers, except for IL-1β, which strongly correlated with TNF- α.

Several studies have already shown the importance of high levels of laboratory markers, such as bilirubin, D-dimer, C-reactive protein, urea, and others, associated with the severity of COVID-19 [21,22,23,24]. Similarly, regarding inflammatory cytokines, high levels of several cytokines, such as IL-1β, IL-2, IL-6, IL-7, IL-8, IL-10, CXCL10/IP-10, MCP-1, and TNF-α have already been described according to the severity of COVID-19 [7,25,26]. Besides confirming previous studies, our study contributes to bringing attention to the higher values of IFN-α, IL-17A, and IL-23 in critically ill COVID-19 individuals, which are less studied than those described above. In a recent study, Smail et al. (2024) showed that high levels of IL-10, IL-23, and TNF-α, as seen in severe and critical cases of COVID-19, were associated with in-hospital mortality [26]. IL-17A plays a crucial role in the pathogenesis of various inflammatory and autoimmune disorders [27,28]. In COVID-19, the IL-17A is elevated in a few studies and is mainly associated with the severity of COVID-19, a finding corroborated by our study. Shibabaw et al. (2020) show that the synergistic interaction of IL-17A and IL-6 is the central player in developing pulmonary fibrosis and an impaired respiratory system in COVID-19 [29].

Concerning COVID-19 in PLWH, it is essential to point out that the inpatients included in our study were younger than the COVID-19 group but heavily immunosuppressed. A limited number of these individuals were not aware of their HIV status or were receiving ART. Although no statistically significant differences were observed between the two groups regarding hospital discharge or mortality rates (Table 2), it is noteworthy that active tuberculosis was more prevalent in the PLWH/COVID-19 group. According to the WHO statement, HIV infection is a risk factor for severe or critical COVID-19 at hospital admission and for in-hospital mortality [30]. However, based on a meta-analysis of epidemiological studies, Favara et al. (2022) concluded that HIV infection did not affect the risk of death of COVID-19 patients [31]. Possibly in consequence of the heavy immunosuppression in the PLWH/COVID-19 group included in our study, we observed lower levels of almost all inflammatory parameters analyzed, compared to the COVID-19 group, suggesting the hyper-inflammation, with increased release of cytokines or other inflammatory markers, might not be playing a role in the pathogeny of PLWH/COVID-19 individuals. This profile differs from those observed in different studies, including immune-competent PLWH with COVID-19, according to or not with the COVID-19 clinical profile [32]. In a recent study conducted by Anzurez et al. (2024), no differences in cytokines levels were observed according to the COVID-19 clinical profiles among PLWH, compared to the higher levels observed among those with severe COVID-19 without HIV infection [32]. Moreover, strong associations between the cytokines IFN-α/TNF-α and others observed in the COVID-19 group were lost in PLWH [32].

Some limitations of this study should be noted, mainly concerning the analysis of inflammatory mediators over time. Many of the individuals analyzed will require long periods of intensive care before clinical improvement. It would, therefore, be interesting to monitor the inflammatory response throughout the hospital stay and elucidate any differences in the inflammatory profile. Regarding the impact of vaccination against COVID-19 on the individuals analyzed, it is essential to note that the participants included in this study had not been vaccinated at the time of collecting the material for analysis.

## 5. Conclusions

This study significantly advances our understanding of COVID-19 severity by evaluating a comprehensive panel of cytokines and clinical markers, confirming the robust association between elevated levels of IL-1β, IL-6, IL-8, IL-10, IL-17A, IL-23, TNF-α, and IFN-α with critical disease outcomes. Moreover, it underscores the potential role of lesser-explored cytokines, such as IFN-α, IL-17A, and IL-23, in severe COVID-19. The inclusion of people living with HIV (PLWH) offers a unique perspective on the interplay between HIV and COVID-19, revealing that, despite their immunosuppressed state, PLWH exhibited lower levels of inflammatory markers and a higher prevalence of active tuberculosis without an associated increase in mortality or hospital discharge rates. These findings suggest that hyper-inflammation may not be a key driver of COVID-19 pathogenesis in PLWH, highlighting the complex immunological dynamics between HIV and COVID-19 and offering crucial insights for personalized therapeutic approaches.

## Figures and Tables

**Table 1:** Sociodemographic and clinical features of COVID-19 individuals compared with COVID-19 severity.

Features		Overall N = 134	Moderate N = 30	Severe N = 67	Critical N = 32	*p*-Value ^a^
Sociodemographic						
Gender *; n (%)	Female	60 (44.8%)	15 (50%)	29 (43.3%)	13(40.6%)	0.742
	Male	74 (55.2%)	15 (50%)	38 (56.7%)	19 (59.4%)	
Skin Color; n (%)	White	21 (15.7%)	4 (13.3%)	8 (11.9%)	8 (25%)	NC
	Brown	91 (67.9%)	21 (70%)	46 (68.7%)	22 (68.8%)	
	Black	10 (7.5%)	4 (13.3%)	5 (7.5%)	1 (3.1%)	
	Others	2 (1.5%)	0 (0%)	2 (3%)	0 (0%)	
Age; n (IQR)		58.22 (IQR = 21.3)	60.27 (IQR = 18.61)	55.49 (IQR = 19.8)	65.85 (IQR = 25.61)	0.104
	(18–40]	18 (13.8%)	4 (13.8%)	6 (9.1%)	6 (19.4%)	0.059
	(40–60]	49 (37.7%)	9 (31%)	33 (50%)	6 (19.4%)	
	(60–80]	57 (43.8%)	13 (44.8%)	26 (39.4%)	17 (54.8%)	
	(80–90]	6 (4.6%)	3 (10.3%)	1 (1.5%)	2 (6.5%)	
Schooling; n (%)	University education	9 (8.8%)	1 (4%)	3 (6.4%)	4 (16%)	0.243
	High school	44 (43.1%)	9 (36%)	20 (42.6%)	13 (52%)	
	Low Education	49 (48%)	15 (60%)	24 (51.1%)	8 (32%)	
**Comorbidities**						
HAS; n (%)	No	74 (55.2%)	15 (50%)	39 (58.2%)	15 (46.9%)	0.52
	Yes	60 (44.8%)	15 (50%)	28 (41.8%)	17 (53.1%)	
Diabetes Mellitus (DM); n (%)	No	94 (70.1%)	23 (76.7%)	41 (61.2%)	26 (81.2%)	0.082
	Yes	40 (29.9%)	7 (23.3%)	26 (38.8%)	6 (18.8%)	
Cardiac Insufficiency; n (%)	No	129 (96.3%)	28 (93.3%)	66 (98.5%)	30 (93.8%)	0.344
	Yes	5 (3.7%)	2 (6.7%)	1 (1.5%)	2 (6.2%)	
COPD; n (%)	No	124 (92.5%)	29 (96.7%)	64 (95.5%)	27 (84.4%)	0.084
	Yes	10 (7.5%)	1 (3.3%)	3 (4.5%)	5 (15.6%)	
Coronary Artery Disease; n (%)	No	133 (99.3%)	29 (96.7%)	67 (100%)	32 (100%)	0.19
	Yes	1 (0.7%)	1 (3.3%)	0 (0%)	0 (0%)	
Obesity; n (%)	No	113 (84.3%)	24 (80%)	58 (86.6%)	27 (84.4%)	0.711
	Yes	21 (15.7%)	6 (20%)	9 (13.4%)	5 (15.6%)	
Active Tuberculosis; n (%)	No	129 (96.3%)	30 (100%)	66 (98.5%)	30 (93.8%)	0.213
	Yes	5 (3.7%)	0 (0%)	1 (1.5%)	2 (6.2%)	
HIV infection *	No	112 (83.6%)	29 (96.7%)	51 (76.1%)	31 (96.9%)	0.003
	Yes	22 (16.4%)	1 (3.3%)	16 (23.9%)	1 (3.1%)	
**Symptoms**						
Fever; n (%)	No	64 (47.8%)	15 (50%)	26 (38.8%)	19 (59.4%)	0.144
	Yes	70 (52.2%)	15 (50%)	41 (61.2%)	13 (40.6%)	
Cough; n (%)	No	49 (36.6%)	8 (26.7%)	21 (31.3%)	18 (56.2%)	0.025
	Yes	85 (63.4%)	22 (73.3%)	46 (68.7%)	14 (43.8%)	
Chest Pain; n (%)	No	116 (86.6%)	27 (90%)	55 (82.1%)	30 (93.8%)	0.232
	Yes	18 (13.4%)	3 (10%)	12 (17.9%)	2 (6.2%)	
Coryza; n (%)	No	125 (93.3%)	28 (93.3%)	63 (94%)	30 (93.8%)	0.991
	Yes	9 (6.7%)	2 (6.7%)	4 (6%)	2 (6.2%)	
Dyspneia; n (%)	No	29 (21.6%)	4 (13.3%)	14 (20.9%)	8 (25%)	0.507
	Yes	105 (78.4%)	26 (86.7%)	53 (79.1%)	24 (75%)	
Odynophagy; n (%)	No	132 (98.5%)	29 (96.7%)	66 (98.5%)	32 (100%)	0.568
	Yes	2 (1.5%)	1 (3.3%)	1 (1.5%)	0 (0%)	
Anosmia; n (%)	No	122 (91%)	28 (93.3%)	58 (86.6%)	31 (96.9%)	0.218
	Yes	12 (9%)	2 (6.7%)	9 (13.4%)	1 (3.1%)	
Loss Of Taste; n (%)	No	122 (91%)	29 (96.7%)	57 (85.1%)	31 (96.9%)	0.073
	Yes	12 (9%)	1 (3.3%)	10 (14.9%)	1 (3.1%)	
Diarrhea; n (%)	No	123 (91.8%)	27 (90%)	61 (91%)	31 (96.9%)	0.521
	Yes	11 (8.2%)	3 (10%)	6 (9%)	1 (3.1%)	
Abdominal Pain; n (%)	No	131 (97.8%)	30 (100%)	65 (97%)	31 (96.9%)	0.627
	Yes	3 (2.2%)	0 (0%)	2 (3%)	1 (3.1%)	
Nausea; n (%)	No	131 (97.8%)	28 (93.3%)	66 (98.5%)	32 (100%)	0.178
	Yes	3 (2.2%)	2 (6.7%)	1 (1.5%)	0 (0%)	
Headache; n (%)	No	120 (89.6%)	28 (93.3%)	57 (85.1%)	30 (93.8%)	0.302
	Yes	14 (10.4%)	2 (6.7%)	10 (14.9%)	2 (6.2%)	
Myalgia; n (%)	No	110 (82.1%)	25 (83.3%)	53 (79.1%)	27 (84.4%)	0.781
	Yes	24 (17.9%)	5 (16.7%)	14 (20.9%)	5 (15.6%)	
**Severity status**						
Oxygen supplementation or ventilatory support	No	18 (13.4%)	2 (6.7%)	11 (16.4%)	1 (3.1%)	0.097
	Yes	116 (86.6%)	28 (93.3%)	56 (83.6%)	31 (96.9%)	
WHO scale; n		8	5	8	9	NC
Glasgow scale cat	(2.9,9]	6 (5.6%)	0 (0%)	2 (3.4%)	4 (21.1%)	0.005
	(9,13]	8 (7.5%)	3 (12.5%)	2 (3.4%)	3 (15.8%)	
	(13,15.1]	93 (86.9%)	21 (87.5%)	55 (93.2%)	12 (63.2%)	
SOFA cat	(0,10]	127 (95.5%)	30 (100%)	64 (97%)	28 (87.5%)	0.044
	(10,12.1]	6 (4.5%)	0 (0%)	2 (3%)	4 (12.5%)	
SAPS-III cat	(30.9,57]	98 (73.7%)	25 (83.3%)	51 (77.3%)	17 (53.1%)	0.014
	(57,98.1]	35 (26.3%)	5 (16.7%)	15 (22.7%)	15 (46.9%)	
**Clinical Parameters of HIV-1 infection**						
CD4 counts, cells/mm3 (IQR)		64 (IQR = 239)	19 (IQR = 0)	68.5 (IQR = 234)	385 (IQR = 0)	0.487
CD8 counts, cells/mm3 (IQR)		514 (IQR = 351)	520 (IQR = 0)	467.5 (IQR = 488.75)	719 (IQR = 0)	0.74
Viral load, HIV RNA copies/mL (IQR)		92,151 (IQR = 628,649.75)	695 (IQR = 0)	98,311 (IQR = 624,932.5)	6938 (IQR = 0)	0.187
Viral load Log_10_/mL		4.96 (IQR = 1.94)	2.84 (IQR = 0)	4.99 (IQR = 1.4)	3.84 (IQR = 0)	0.187

Data are expressed as absolute (relative) frequencies for nominal variables and as medians and interquartile ranges (IQRs) for continuous numerical variables. ^a^ *p*-values were calculated using chi-square tests for nominal variables and Kruskall–Wallis tests for continuous–numerical variables; *p*-values < 0.05 were considered significant. Abbreviations: HAS: Systemic Arterial Hypertension. N: number of individuals in each group. NC: not calculated. COPD: Chronic obstructive pulmonary disease. WHO: World Health Organization. SOFA: Sequential Organ Failure Assessment. SAPS III: Simplified Acute Physiology Score III. Cat: Category of small categories. Glasgow scale cat (2.9,9): severe trauma; (9,13): moderate trauma; (13,15.1): mild trauma/normal. SOFA cat (0,10]: corresponded to a mortality rate of 60%; (10,12.1]: corresponded to mortality rate >90%. SAPS3 cat (30,57]: corresponded to mortality of <3%; (57,98.1]: corresponded to a mortality rate of >70%. * The COVID-19 severity classification was not possible for four PLWH: two females and two males.

**Table 2:** Sociodemographic and clinical features of COVID-19 individuals compared with COVID/PLWH individuals.

Features		Overall N = 134	COVID-19 N = 112	COVID/PLWH N = 22	*p*-Value ^a^
Sociodemographic					
Gender; n (%)	Female	60 (44.8%)	51 (45.5%)	9 (40.9%)	0.869
	Male	74 (55.2%)	61 (54.5%)	13 (59.1%)	
Skin Color; n (%)	White	21 (15.7%)	20 (17.9%)	1 (4.5%)	NC
	Brown	91 (67.9%)	74 (66.1%)	17 (77.3%)	
	Black	10 (7.5%)	9 (8%)	1 (4.5%)	
	Others	2 (1.5%)	2 (1.8%)	0 (0%)	
Age; n (IQR)		58.22 (IQR = 21.3)	62.61 (IQR = 19.37)	44.37 (IQR = 17.84)	<0.001
	(18–40]	18 (13.8%)	9 (8.2%)	9 (45%)	<0.001
	(40–60]	49 (37.7%)	41 (37.3%)	8 (40%)	
	(60–80]	57 (43.8%)	54 (49.1%)	3 (15%)	
	(80–90]	6 (4.6%)	6 (5.5%)	0 (0%)	
Schooling; n (%)	University education	9 (8.8%)	8 (9.1%)	1 (7.1%)	0.521
	High school	44 (43.1%)	36 (40.9%)	8 (57.1%)	
	Low Education	49 (48%)	44 (50%)	5 (35.7%)	
**Comorbidities**					
HAS; n (%)	No	74 (55.2%)	56 (50%)	18 (81.8%)	0.012
	Yes	60 (44.8%)	56 (50%)	4 (18.2%)	
Diabetes Mellitus (DM); n (%)	No	94 (70.1%)	77 (68.8%)	17 (77.3%)	0.587
	Yes	40 (29.9%)	35 (31.2%)	5 (22.7%)	
Cardiac Insufficiency; n (%)	No	129 (96.3%)	107 (95.5%)	22 (100%)	0.693
	Yes	5 (3.7%)	5 (4.5%)	0 (0%)	
COPD; n (%)	No	124 (92.5%)	104 (92.9%)	20 (90.9%)	1
	Yes	10 (7.5%)	8 (7.1%)	2 (9.1%)	
Coronary Artery Disease; n (%)	No	133 (99.3%)	111 (99.1%)	22 (100%)	1
	Yes	1 (0.7%)	1 (0.9%)	0 (0%)	
Obesity; n (%)	No	113 (84.3%)	93 (83%)	20 (90.9%)	0.543
	Yes	21 (15.7%)	19 (17%)	2 (9.1%)	
Active Tuberculosis; n (%)	No	129 (96.3%)	111 (99.1%)	18 (81.8%)	0.001
	Yes	5 (3.7%)	1 (0.9%)	4 (18.2%)	
**Symptoms**					
Fever; n (%)	No	64 (47.8%)	55 (49.1%)	9 (40.9%)	0.638
	Yes	70 (52.2%)	57 (50.9%)	13 (59.1%)	
Cough; n (%)	No	49 (36.6%)	40 (35.7%)	9 (40.9%)	0.826
	Yes	85 (63.4%)	72 (64.3%)	13 (59.1%)	
Chest Pain; n (%)	No	116 (86.6%)	95 (84.8%)	21 (95.5%)	0.32
	Yes	18 (13.4%)	17 (15.2%)	1 (4.5%)	
Coryza; n (%)	No	125 (93.3%)	106 (94.6%)	19 (86.4%)	0.341
	Yes	9 (6.7%)	6 (5.4%)	3 (13.6%)	
Dyspnea; n (%)	No	29 (21.6%)	20 (17.9%)	9 (40.9%)	0.034
	Yes	105 (78.4%)	92 (82.1%)	13 (59.1%)	
Odynophagy; n (%)	No	132 (98.5%)	110 (98.2%)	22 (100%)	1
	Yes	2 (1.5%)	2 (1.8%)	0 (0%)	
Anosmia; n (%)	No	122 (91%)	100 (89.3%)	22 (100%)	0.23
	Yes	12 (9%)	12 (10.7%)	0 (0%)	
Loss Of Taste; n (%)	No	122 (91%)	100 (89.3%)	22 (100%)	0.23
	Yes	12 (9%)	12 (10.7%)	0 (0%)	
Diarrhea; n (%)	No	123 (91.8%)	103 (92%)	20 (90.9%)	1
	Yes	11 (8.2%)	9 (8%)	2 (9.1%)	
Abdominal Pain; n (%)	No	131 (97.8%)	109 (97.3%)	22 (100%)	1
	Yes	3 (2.2%)	3 (2.7%)	0 (0%)	
Nausea; n (%)	No	131 (97.8%)	109 (97.3%)	22 (100%)	1
	Yes	3 (2.2%)	3 (2.7%)	0 (0%)	
Headache; n (%)	No	120 (89.6%)	99 (88.4%)	21 (95.5%)	0.543
	Yes	14 (10.4%)	13 (11.6%)	1 (4.5%)	
Myalgia; n (%)	No	110 (82.1%)	93 (83%)	17 (77.3%)	0.734
	Yes	24 (17.9%)	19 (17%)	5 (22.7%)	
**Severity status**					
Oxygen supplementation or ventilatory support	No	18 (13.4%)	10 (8.9%)	8 (36.4%)	0.002
	Yes	116 (86.6%)	102 (91.1%)	14 (63.6%)	
WHO scale		8 (IQR = 0)	8 (IQR = 3.5)	8 (IQR = 0)	0.906
WHO scale cat	moderate	30 (23.3%)	29 (26.1%)	1 (5.6%)	0.106
	severe/critical	99 (76.7%)	82 (73.9%)	17 (94.4%)	
Glasgow scale cat	(2.9,9]	6 (5.6%)	5 (5.7%)	1 (5%)	0.361
	(9,13]	8 (7.5%)	8 (9.2%)	0 (0%)	
	(13,15.1]	93 (86.9%)	74 (85.1%)	19 (95%)	
SOFA cat	(0,10]	127 (95.5%)	106 (95.5%)	21 (95.5%)	1
	(10,12.1]	6 (4.5%)	5 (4.5%)	1 (4.5%)	
SAPS-III cat	(30.9,57]	98 (73.7%)	81 (73%)	17 (77.3%)	
	(57,98.1]	35 (26.3%)	30 (27%)	5 (22.7%)	
Outcomes; n (%)	discharge	80 (59.7%)	66 (58.9%)	14 (63.6%)	0.862
	death	54 (40.3%)	46 (41.1%)	8 (36.4%)	

Data are expressed as absolute (relative) frequencies for nominal variables and as medians and interquartile ranges (IQRs) for continuous numerical variables. ^a^ *p*-values were calculated using chi-square tests for nominal variables and Mann–Whitney U tests for continuous–numerical variables; *p*-values < 0.05 were considered significant. Abbreviations: HAS: Systemic Arterial Hypertension. N: number of individuals in each group. NC: not calculated. COPD: Chronic obstructive pulmonary disease. WHO: World Health Organization. SOFA: Sequential Organ Failure Assessment. SAPS III: Simplified Acute Physiology Score III. Cat: Category of small categories. Glasgow scale cat (0,9): severe trauma; (9,13): moderate trauma; (13,15.1): mild trauma/normal. SOFA cat (0,10]: corresponded to a mortality rate of 60%; (10,12.1]: corresponded to mortality rate >90%. SAPS3 cat (30,57]: corresponded to mortality of <3%; (57,98.1]: corresponded to a mortality rate of >70%.

## Data Availability

The data that support the findings of this study are available upon request from the corresponding author. The data are not publicly available due to privacy or ethical restrictions.

## Supplementary Materials

The following supporting information can be downloaded at https://www.mdpi.com/article/10.3390/v17010091/s1, Figure S1: Comparison of laboratory markers levels of COVID-19 individuals (in red) compared with COVID/PLWH individuals (in blue). The graphs represent the concentration measures of (A) Alanine aminotransferase, (B) Aspartate aminotransferase. The sampling distributions of the data are represented in the form of a colored box plot. In black, the center circle represents each group’s expected mean marginal effect, estimated from linear fixed effects models including sex, age, and days since the first symptoms as confounding variables. The horizontal bars represent the 95% confidence intervals of the expected mean marginal effects by group. * *p* < 0.05; ** *p* < 0.01; Table S1: Baseline sampled data of laboratory clinical markers and cytokine plasma levels of all study participants. Data are expressed as absolute (relative) frequencies for nominal variables and as medians and interquartile range ranges (IQRs) for continuous numerical variables. a *p*-value were calculated using Chi-square tests for nominal variables, and Mann-Whitney U tests for continuous numerical variables. *p*-values < 0.05 were considered significant. Abbreviations: N: number of individuals in each group; ALT: alanine aminotransferase; AST: aspartate aminotransferase; CRP: C-reactive protein; ESR: erythrocyte sedimentation rate; HBA1C: hemoglobin A1c; PTA: prothrombin time activity; PTT: partial thromboplastin time.
