# Supplementary material for: COVID-19 and HIV: Clinical Outcomes and Inflammatory Markers in a Cohort from a Reference Hospital in Rio de Janeiro, Brazil
Source: Viruses. 2025 Jan 13;17(1):91. doi: 10.3390/v17010091 (PMC11769093; doi:10.3390/v17010091)
Supplement: Supplementary file 1 [file viruses-17-00091-s001.zip › viruses-3403947-supplementary.pdf]

Supplementary Material

Table S1: Baseline sampled data of laboratory clinical markers and cytokine plasma levels of all study participants.

Features	Overall N=134
**Leukocytes**	10260 (IQR=7710)
**Lymphocytes**	1121 (IQR=1122)
**Platelets**	258 (IQR=127.5)
**Creatinine**	1.05 (IQR=0.62)
**Amylase**	57 (IQR=43.5)
**Lipase**	36 (IQR=34)
**ALT**	36 (IQR=32)
**AST**	38 (IQR=30)
**Bilirubin**	0.48 (IQR=0.39)
**DDimer**	1.95 (IQR=5.9)
**CRP**	11.05 (IQR=13.88)
**ESR**	90 (IQR=60)
**Procalcitonin**	0.27 (IQR=0.92)
**Ferritin**	525.9 (IQR=743.5)
**Troponin**	0.03 (IQR=0)
**HBA1C**	6.1 (IQR=1.8)
**PTA**	1.04 (IQR=0.18)
**PTT**	27 (IQR=6.6)
**Urea**	52.5 (IQR=55.2)
**IP-10**	44.17 (IQR=231.82)
**IL-8**	102.16 (IQR=507.12)
**IL-10**	156.27 (IQR=260.34)
**TNF-α**	1266.77 (IQR=1778.45)
**IFN-α**	19.34 (IQR=27.59)
**IL-33**	139.25 (IQR=277.59)
**IL-17F**	2.31 (IQR=2.35)
**IL-17AF**	226.16 (IQR=336.03)
**IL-1β**	114.78 (IQR=332.37)
**IL-12p70**	24 (IQR=19.24)
**IL-17A**	53.78 (IQR=90.44)
**IFN-γ**	186.15 (IQR=238.19)
**IL-23**	681.39 (IQR=1126.78)
**IL-18**	698.97 (IQR=899.69)
**IL-6**	41.52 (IQR=116.54)

Data are expressed as absolute (relative) frequencies for nominal variables and as medians and interquartile range ranges (IQRs) for continuous numerical variables. [a]*P*-value were calculated using Chi-square tests for nominal variables, and Mann-Whitney U tests for continuous numerical variables. P-values < 0.05 were considered significant. Abbreviations: N: number of individuals in each group; ALT: alanine aminotransferase; AST: aspartate aminotransferase; CRP: C-reactive protein; ESR: erythrocyte sedimentation rate; HBA1C: hemoglobin A1c; PTA: prothrombin time activity; PTT: partial thromboplastin time.

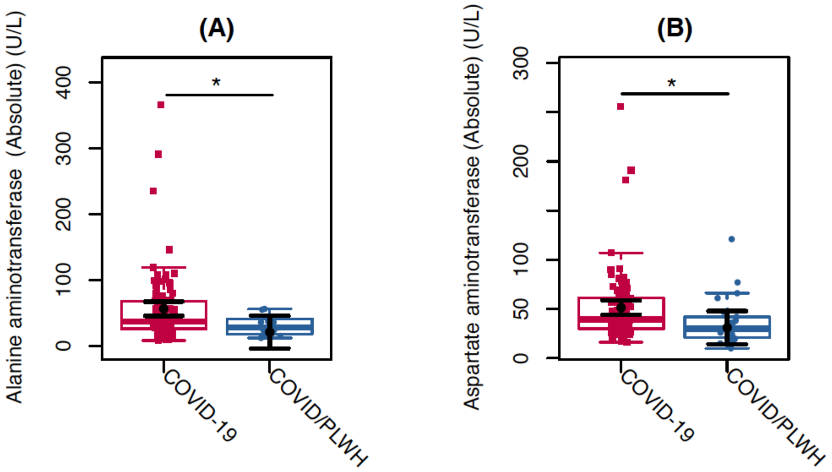

Figure S1: Comparison of laboratory markers levels of COVID-19 individuals (in red) compared with COVID/PLWH individuals (in blue). The graphs represent the concentration measures of (A) Alanine aminotransferase, (B) Aspartate aminotransferase. The sampling distributions of the data are represented in the form of a colored box plot. In black, the center circle represents each group's expected mean marginal effect, estimated from linear fixed effects models including sex, age, and days since the first symptoms as confounding variables. The horizontal bars represent the 95% confidence intervals of the expected mean marginal effects by group. *$p < 0.05$; **$p < 0.01$.
